# IL-21 signaling is essential for optimal host resistance against *Mycobacterium tuberculosis* infection

**DOI:** 10.1038/srep36720

**Published:** 2016-11-07

**Authors:** Matthew G. Booty, Palmira Barreira-Silva, Stephen M. Carpenter, Cláudio Nunes-Alves, Miye K. Jacques, Britni L. Stowell, Pushpa Jayaraman, Gillian Beamer, Samuel M. Behar

**Affiliations:** 1 Department of Microbiology and Physiological Systems, University of Massachusetts Medical School, Worcester, Massachusetts 01655, US; 2 Program in Immunology, Division of Medical Sciences, Harvard Medical School, Boston, Massachusetts 02115, US; 3 Life and Health Sciences Research Institute (ICVS), School of Medicine, University of Minho, Braga, Portugal; 4 ICVS/3B’s, Portuguese Government Associate Laboratory, Braga/Guimarães, Portugal; 5 Department of Infectious Disease and Global Health, Cummings School of Veterinary Medicine, Tufts University, Grafton, MA 01536, US.

## Abstract

IL-21 is produced predominantly by activated CD4^+^ T cells and has pleiotropic effects on immunity via the IL-21 receptor (IL-21R), a member of the common gamma chain (γ_c_) cytokine receptor family. We show that IL-21 signaling plays a crucial role in T cell responses during *Mycobacterium tuberculosis* infection by augmenting CD8^+^ T cell priming, promoting T cell accumulation in the lungs, and enhancing T cell cytokine production. In the absence of IL-21 signaling, more CD4^+^ and CD8^+^ T cells in chronically infected mice express the T cell inhibitory molecules PD-1 and TIM-3. We correlate these immune alterations with increased susceptibility of IL-21R^−/−^ mice, which have increased lung bacterial burden and earlier mortality compared to WT mice. Finally, to causally link the immune defects with host susceptibility, we use an adoptive transfer model to show that IL-21R^−/−^ T cells transfer less protection than WT T cells. These results prove that IL-21 signaling has an intrinsic role in promoting the protective capacity of T cells. Thus, the net effect of IL-21 signaling is to enhance host resistance to *M. tuberculosis*. These data position IL-21 as a candidate biomarker of resistance to tuberculosis.

The human pathogen *Mycobacterium tuberculosis* causes more deaths than any other bacterium. In 2014, there were 9.6 million new cases of TB and 1.5 million deaths^1^. While *M. bovis* BCG is widely used as a vaccine, it’s efficacy in preventing pulmonary TB in adults is highly variable^2^. To combat TB, vaccine development is a strategic priority and clinical testing is underway for new candidates^3^. Unfortunately, critical deficits in our understanding of immunity following infection are impeding progress. Though long-lived immunity to *M. tuberculosis* is clearly T cell-dependent, there remains uncertainty about the molecular basis of T cell mediated clearance of bacteria and there are no validated correlates of protection^4^,^5^,^6^.

The cytokine IL-21 modulates adaptive immunity and affects CD8^+^ T cell responses. Although IL-21 is dispensable for the clearance of acute LCMV infection, mice lacking the IL-21 receptor (IL-21R^−/−^) are unable to control viral replication during chronic LCMV infection^7^,^8^,^9^. This phenotype is attributed to a defective CD8^+^ T cell response, and to IL-21 acting directly on CD8^+^ T cells to promote continuous proliferation during chronic disease. Furthermore during chronic infection, IL-21R^−/−^ CD8^+^ T cells become exhausted, produce less IL-2 and IFNγ, and express more PD-1 inhibitory receptor^7^,^8^.

CD4^+^ T cells are the major source of IL-21 during LCMV infection. Mice lacking CD4^+^ T cells develop severe CD8^+^ T cell exhaustion and are unable to control viral replication following chronic LCMV infection^10^,^11^,^12^. Simply treating CD4^−/−^ knockout (KO) mice with IL-21 rescues CD8^+^ T cell expansion and cytokine production, and reduces viral titers^8^. These studies implicate IL-21 as an essential mediator of CD4^+^ T cell help during infection. Despite these key functions of IL-21, this cytokine has been examined in relatively few chronic infections^13^. In humans, IL-21 prevents T cell exhaustion during HCV infection^14^,^15^. Several clinical studies have detected changes in IL-21 production during active tuberculosis. IL-21 was one of only three cytokines from a panel of 19 that were elevated in pediatric tuberculosis^16^. Another report finds that adults with active tuberculosis have decreased levels of circulating IL-21 when compared to latently infected individuals^17^. At the site of disease, IL-21 mRNA is enriched in lung granulomas of patients with active tuberculosis^18^. Although the role of IL-21 cannot be discerned from these studies, these data implicate IL-21 as a component of the human immune response to *M. tuberculosis*. Based on these data, we sought to determine whether IL-21 served a crucial role in host protection against *M. tuberculosis* infection in the mouse model.

We first test the hypothesis that IL-21 is an important helper cytokine for CD8^+^ T cells during *M. tuberculosis* infection. Using naive CD8^+^ T cells specific for the immunodominant *M. tuberculosis* antigen TB10.4 (EsxH), we discover that IL-21 is essential for efficient CD8^+^ T cell priming. Furthermore, we show that IL-21 signaling maintains T cell numbers and cytokine production. These findings indicate that IL-21 promotes CD4^+^ and CD8^+^ T cell responses throughout infection and its actions are not limited to the late phase of disease. Similar to chronic viral infection, we show that IL-21 signaling is associated with reduced expression of the inhibitory receptors TIM-3 and PD-1. We demonstrate that IL-21 is required for the host to restrain bacterial replication and promote host survival. Finally, we show that IL-21 signaling enhances transfer of protection by T cells. Based on these data, IL-21 joins interferon (IFN)-γ and tumor necrosis factor (TNF) as a T cell-derived cytokines that is critical for host resistance against *M. tuberculosis* infection.

## Results

### IL-21 is produced by CD4^+^ T cells during *M. tuberculosis* infection

**Figure 1:**
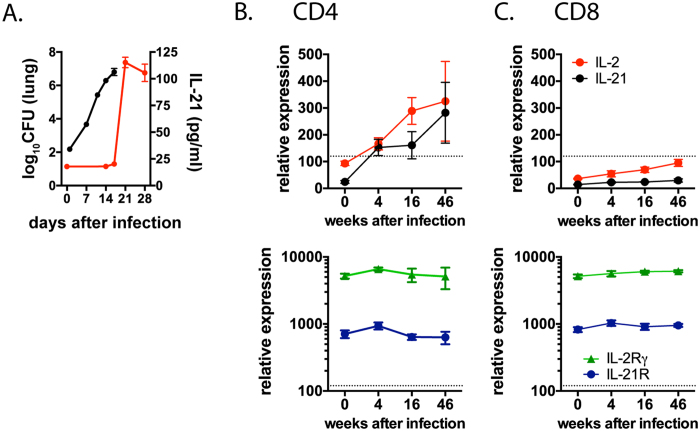
Expression of IL-21 and its receptor components in the lung during *M. tuberculosis* infection. (**A**) IL-21 (red line) in lung homogenate from uninfected (t = 0) or *M. tuberculosis* infected mice and bacillary load (black line, CFU). Expression of IL-2, IL-21, IL-21R, and IL-2Rγ (i.e., γ_c_), by CD4^+^ T cells (B) and CD8^+^ T cells (C) in the lungs of uninfected mice (t = 0) or following infection. Values > 120 (indicated by the dotted line) have a ≥95% probability of true expression.

We measured IL-21 in lung homogenates from *M. tuberculosis* infected mice. A significant increase in IL-21 was detected starting 3 weeks after infection, which is when T cells appear in the lung (Fig. 1a)^19^,^20^,^21^. Thus, instead of being pathogen-triggered, pulmonary IL-21 production is associated with T cell recruitment into the lung. IL-21 transcript levels were induced 10-fold in CD4^+^ T cells following infection; in contrast, little or no transcript was detected in CD8^+^ T cells (Fig. 1b,c). Both the alpha chain (IL-21R) and the common gamma chain (γ_c_) of the IL-21 receptor were constitutively expressed by pulmonary CD4^+^ and CD8^+^ T cells, before and after infection (Fig. 1b,c). Similar results were obtained when T cells from chronically infected mice were anlylsized by Nanostring (Supplemental Fig. 1).Thus, following *M. tuberculosis* infection, CD4^+^ T cells are a source of IL-21, and both CD4^+^ and CD8^+^ T cells express the IL-21R.

### IL-21 promotes CD8^+^ T cell priming during *M. tuberculosis* infection

During *M. tuberculosis* infection, IL-12 promotes CD8^+^ T cell priming, expansion, and the acquisition of effector functions; nevertheless, a *M. tuberculosis*-specific CD8^+^ T cell response is primed even in the absence of IL-12^22^. We considered whether IL-21, a regulator of CD8^+^ T cell expansion, promotes CD8^+^ T cell responses during TB. We asked whether IL-21 supports CD8^+^ T cell priming in the draining LN following infection. As a source of traceable naïve T cells, we used retrogenic mice generated on congenically marked WT (CD45.1) or IL-21R^−/−^ (CD45.2) backgrounds. These retrogenic mice produce CD8^+^ T cells expressing a TCR specific for the immunodominant *M. tuberculosis* epitope TB10.4_4–11_ (TB10Rg)^22^,^23^. Equal numbers of WT and IL-21R^−/−^ TB10Rg CD8^+^ T cells were transferred into recipient mice 7 days after low-dose aerosol *M. tuberculosis* infection, and the ratio of WT and IL-21R^−/−^ TB10Rg CD8^+^ T cells was measured on days 8, 11, 13, and 15 post infection as previously described^22^.

**Figure 2:**
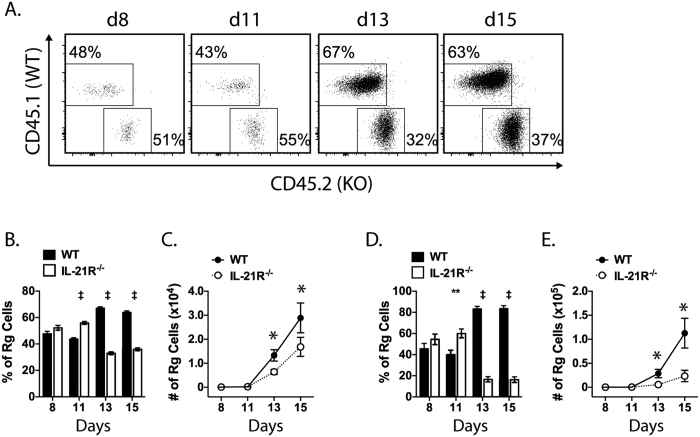
IL-21 signaling is required for efficient CD8^+^ T cell priming and accumulation in the lung. A T cell priming assay was performed by transferring equal numbers of CD45.1^+^ (WT) and CD45.2^+^ (KO, IL-21R^−/−^) TB10Rg3 CD8^+^ T cells into *M. tuberculosis* infected C57BL/6 mice on day 7 post-infection. (**A**) Cytometric plots of WT vs KO TB10Rg3 CD8^+^ T cells in the lung-draining LN of infected mice. The plots are the result of concatenating 5 mice at each time point. (**B**) Composition of the TB10Rg3 CD8^+^ T cell population in the mediastinal LN (MLN) on the indicated days post infection. (**C**) Number of WT or IL-21R^−/−^ TB10Rg3 CD8^+^ T cells in MLNs after transfer. (**D**) Composition of the TB10Rg3 CD8^+^ T cell population in the lung on the indicated days post infection. (**E**) The number of WT or IL-21R^−/−^ TB10Rg3 CD8^+^ T cells in the lung after transfer. Data are representative of two experiments with similar results. Each bar or point represents the mean ± SEM (n = 4–5 mice per group) *P < 0.05, **P < 0.01, ***P < 0.001, ^‡^P < 0.0001.

On day 8, the ratio of WT and IL-21R^−/−^ TB10Rg CD8^+^ T cells was maintained at 1:1 (Fig. 2a,b). Both groups of TB10Rg CD8^+^ T cells underwent significant expansion in the mediastinal LN between days 11–13. At these time points, WT TB10Rg CD8^+^ T cells were overrepresented relative to IL-21R^−/−^ cells and their dominance continued through day 15 (Fig. 2b). As predicted, there were more WT than IL-21R^−/−^ TB10Rg CD8^+^ T cells (Fig. 2c). The number of TB10Rg CD8^+^ T cells was also monitored in the lungs of recipient mice. Just as in the LN, both WT and IL-21R^−/−^ TB10Rg CD8^+^ T cells increased in number during the course of the experiment in the lung, but WT cells expanded more efficiently relative to IL-21R^−/−^ cells (Fig. 2d,e). Thus, IL-21 is necessary for the efficient priming of CD8^+^ T cells in the LN and their accumulation in the lungs *after M. tuberculosis* infection.

### IL-21 signaling is important for T cell expansion in the *M. tuberculosis* infected lung

The availability of large numbers of naïve TB10Rg CD8^+^ T cells makes possible the detection of the earliest events of T cell priming. However, this approach is limited to a single TCR specific for a single epitope. To determine how IL-21 signaling affects polyclonal T cell responses, we made 1:1 mixed bone marrow chimeras (MBMCs) using donor BM from IL-21R^−/−^ (CD45.2^+^ IL-21R^−/−^) or WT (CD45.1^+^ IL-21R^+/+^) mice. This strategy, which we previously validated in the low-dose aerosol model of *M. tuberculosis* infection^22^, has the key advantage of exposing both WT and IL-21R^−/−^ T cells to the same inflammatory environment and bacterial burden during infection.

**Figure 3:**
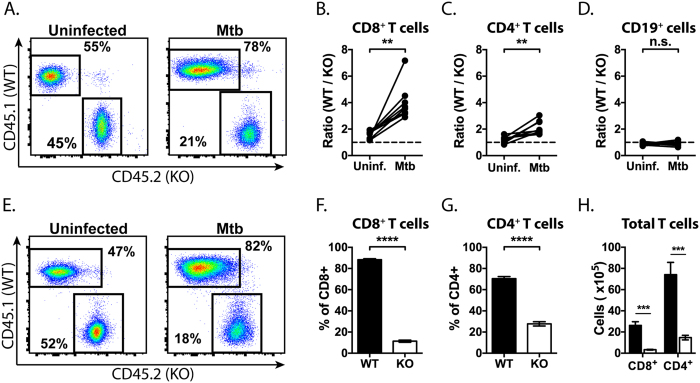
IL-21 signaling is important for T cell expansion during *M. tuberculosis* infection. (**A**) Representative flow cytometric plots showing the distribution of CD45.1^+^ (WT) and CD45.2^+^ (KO, IL-21R^−/−^) among CD8^+^ T cells in blood from the same MBMC mouse before and four weeks after *M. tuberculosis* infection. The ratio of WT to IL-21R^−/−^ CD8^+^ T cells (**B**) CD4^+^ T cells (**C**) and B cells (**D**) in matched blood from the same mice before and 4 weeks after *M. tuberculosis* infection. (**E**) Representative flow cytometric plots showing the distribution of CD45.1^+^ (WT) and CD45.2^+^ (IL-21R^−/−^) among CD8^+^ T cells in lung from an uninfected or *M. tuberculosis* infected MBMC mouse. The distribution of CD45.1^+^ (WT) and CD45.2^+^ (IL-21R^−/−^) among CD8^+^ T cells (**F**) or CD4^+^ T cells (**G**) in lung from MBMC mice infected for four weeks with *M. tuberculosis*. (**H**) Total number of WT (filled) or IL-21R^−/−^ (open) CD8^+^ or CD4^+^ T cells per lung in MBMC mice. Each bar or point represents the mean ± SEM (n = 8 mice per group) *P < 0.05, **P < 0.01, ***P < 0.001, ****P < 0.0001. Data are representative of three independent experiments.

Following reconstitution, uninfected chimeras had similar numbers of WT and KO CD8^+^ T cells, CD4^+^ T cells, and B cells in peripheral blood (Fig. 3a–d). Thus, defective IL-21 signaling did not significantly alter T cell development or lymphocyte homeostasis in uninfected mice. Once hematopoietic reconstitution was established, the chimeras were infected with aerosolized *M. tuberculosis* and analyzed four weeks later, during the peak of the T cell response to *M. tuberculosis.* After infection, WT CD4^+^ and CD8^+^ T cells, but not B cells, were overrepresented in blood relative to IL-21R^−/−^ cells (Fig. 3a–d). This was also true in the lungs of infected mice, where the percentage and number of WT CD4^+^ and CD8^+^ T cells were increased relative to their IL-21R^−/−^ counterparts (Fig. 3e–h). As in peripheral blood, the skewing was more extreme among CD8^+^ T cells than CD4^+^ T cells (Fig. 3f,g). These data show that IL-21 signaling is necessary for the expansion and accumulation of T cells in the lung during TB.

### IL-21 signaling is crucial for T cell cytokine production during *M. tuberculosis* infection

**Figure 4:**
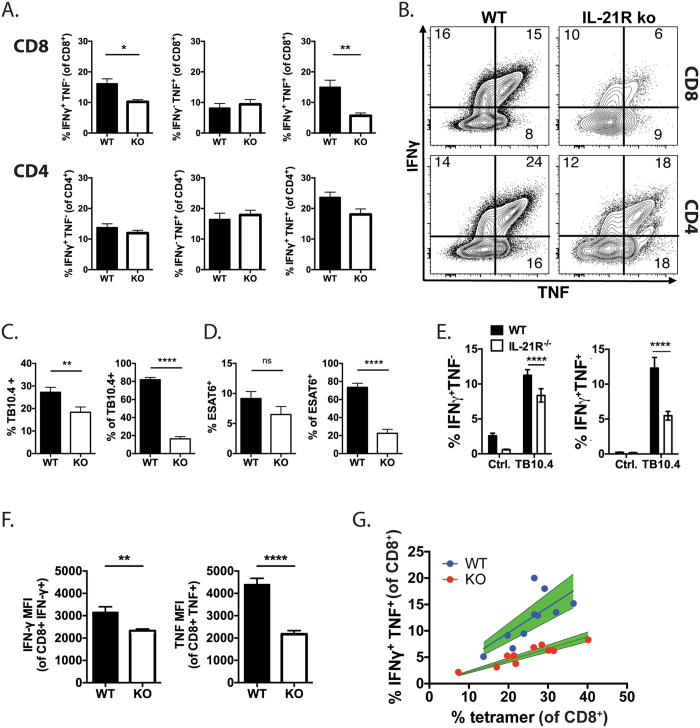
IL-21 signaling promotes cytokine production by T cells during *M. tuberculosis* infection. (**A**) Frequency of WT or IL-21R^−/−^ (KO) CD8^+^ (top row) or CD4^+^ (bottom) T cells from the lungs of infected MBMC mice producing only IFNγ (left), only TNF (middle), or both (right) after stimulation with anti-CD3/28 mAbs. The results are representative of four independent infections (28 mice). (**B**) Representative flow cytometric plots showing IFNγ and TNF production by WT or IL-21R^−/−^ CD8^+^ or CD4^+^ T cells from the lungs of infected MBMC mice after stimulation with anti-CD3/28 mAbs. (**C**) Left: percentages of TB10-specific CD8^+^ T cells (% tetramer^+^) within CD45.1^+^ (WT) or CD45.2^+^ (KO, IL-21R^−/−^) CD8^+^ T cell populations in the lung of infected MBMC mice. Right: percentages of TB10-specific CD8^+^ T cells (% of tetramer^+^) that are CD45.1^+^ (WT) or CD45.2^+^ (KO, IL-21R^−/−^). Pooled data from 4 independent experiments (28 mice). (**D**). Left: Percent of ESAT6-specific CD4^+^ T cells (% tetramer^+^) within CD45.1^+^ (WT) or CD45.2^+^ (KO, IL21R^−/−^) populations in the lung of infected MBMC mice. Right: Percent of ESAT6-specific CD4^+^ T cells (% of tetramer^+^) that are CD45.1^+^ (WT) or CD45.2^+^ (KO, IL-21R^−/−^). Pooled data from 2 independent experiments (10 mice). (**E**) Frequency of WT (closed) or IL-21R^−/−^ (open) CD8^+^ T cells from the lungs of infected MBMC mice that produce only IFNγ (left), or TNF and IFNγ (right) after stimulation with nothing (Ctrl) or TB10.4_4–11_ peptide (TB10.4). (**F**) Intracellular MFI of IFNγ (left) or TNF (right) in cytokine positive cells from the lungs of infected MBMC mice after stimulation with TB10.4_4–11_ peptide. (**G**) Correlation between the frequency of WT (blue) or IL-21R^−/−^ (red) TB10-specific CD8^+^ T cells from the lungs of infected MBMC mice (n = 10) and their production of IFNγ and TNF after stimulation with TB10.4_4–11_ peptide. Green shading, 95% confidence intervals. Each bar represents the mean ± SEM. *P < 0.05, **P < 0.01, ***P < 0.001, ****P < 0.0001. Data are representative of three independent experiments (n = 5, n = 8, or n = 10).

The acquisition of T cell cytokine production was analyzed using infected MBMC mice. Lung cells were stimulated *ex vivo* with anti-CD3/CD28 mAbs, and the frequency of WT or KO CD4^+^ or CD8^+^ T cells that made IFNγ, TNF or both, was determined (Fig. 4a, top and bottom rows, respectively). IL-21R^−/−^ CD4^+^ and CD8^+^ T cells had a diminished capacity to produce IFNγ (Fig. 4a, left). Furthermore, the proportion of IL-21R^−/−^ CD4^+^ or CD8^+^ T cells capable of producing both IFNγ and TNF was also reduced. The magnitude of T cell dysfunction was greatest for IL-21R^−/−^ CD8^+^ T cells (Fig. 4b). Therefore, we next tested whether the antigen-specific CD8^+^ T responses were altered.

There were greater numbers of WT than IL-21R^−/−^ TB10-specific CD8^+^ T cells in the lungs of infected MBMC mice, which is a confounder in the analysis of antigen-specific T cell function (Fig. 3f,c). However, the frequency of TB10-specific CD8^+^ T cells within each genotype was similar, allowing for the normalization of the WT vs. IL-21R^−/−^ CD8^+^ T cell response (Fig. 4d). Lung cells obtained from infected MBMC mice were stimulated with TB10_4–11_ peptide *ex vivo*. A higher proportion of WT CD8^+^ T cells produced IFNγ and TNF than IL-21R^−/−^ CD8^+^ T cells (Fig. 4e). WT CD8^+^ T cells also produced significantly more TNF, and to a lesser degree IFNγ, per cell, based on median fluorescence intensity (Fig. 4f). These results were confirmed by correlating the frequency of TB10-specific CD8^+^ T cells (e.g., tetramer^+^) and the CD8^+^ T cell cytokine response to stimulation by TB10_4–11_ (e.g., IFNγ^+^TNF^+^). WT CD8^+^ T cells were proportionally better at cytokine production than the IL-21R^−/−^ CD8^+^ T cells (Fig. 4h). Thus, IL-21 signaling maintains T cell cytokine production during *M. tuberculosis* infection.

### Loss of IL-21 signaling is associated with increased T cell expression of the TIM-3 and PD-1 inhibitory receptors

The loss of IFNγ/TNF dual-producing T cells is characteristic of T cell exhaustion during chronic viral infection and IL-21 is capable of preventing T cell exhaustion^7^,^9^,^24^,^25^. Therefore, we assessed whether the expression of the PD-1 and TIM-3 inhibitory receptors by pulmonary T cells was altered during *M. tuberculosis* infection in WT and IL-21R^−/−^ mice. We first looked 4 weeks after infection in MBMC or intact WT and IL-21R^−/−^ mice. However, at this early time point, which corresponds to the peak of the T cell response, little PD-1 or TIM-3 was expressed by T cells (data not shown). Since we had previously shown that T cells express abundant cell surface TIM-3 during chronic infection^26^, we next looked 16 weeks post-infection.

**Figure 5:**
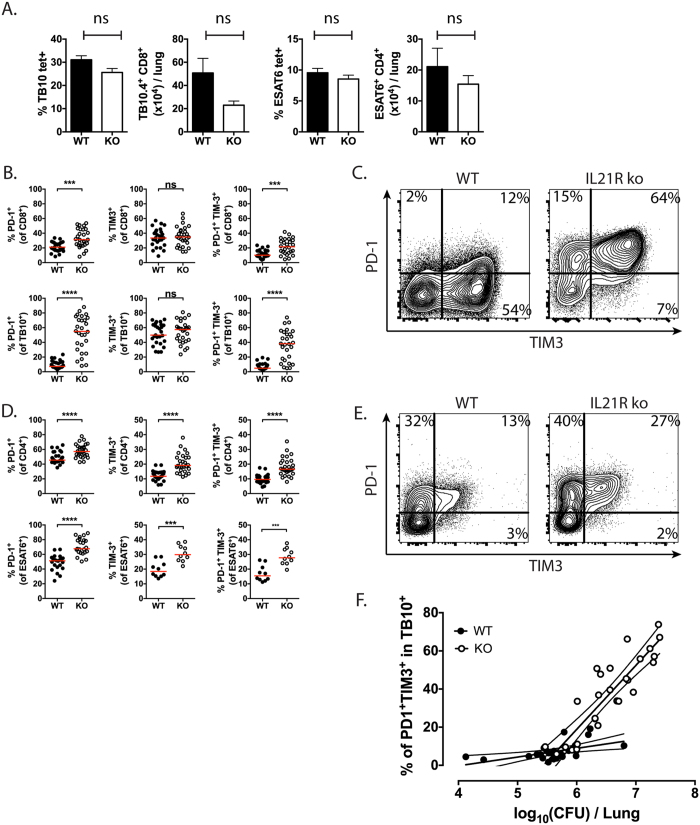
Loss of IL-21R signaling is associated with greater expression of the inhibitory T cell receptors TIM-3 and PD-1. (**A**) The frequency and absolute numbers of ESAT6-specific CD4^+^ T cells (% ESAT6 tet^+^/CD4^+^) or TB10.4-specific CD8^+^ T cells (% TB10 tet^+^/CD8^+^) in the lungs of WT (closed bars) or IL-21R^−/−^ (open bars) mice, 16-20 weeks after *M. tuberculosis* infection. The differences between WT and KO were not significant. Data is pooled from 4 (CD4) or 5 (CD8) experiments with a total of 18 or 23 mice/group analyzed. (**B**) Percentage of CD8^+^ T cells (top row) or TB10.4-specific CD8^+^ T cells (bottom row) that express PD-1 (left), TIM-3 (center), or both PD-1 and TIM-3 (right) in the lungs of WT or IL-21R^−/−^ mice infected with *M. tuberculosis* for 16 weeks. Data is pooled from 4 experiments (n = 5, n = 5, n = 5, or n = 8/group). (**C**) Representative flow cytometric plots showing the expression of PD-1 and TIM-3 by TB10.4-specific CD8^+^ T cells from the lungs of WT or IL-21R^−/−^ mice 16 weeks after *M. tuberculosis* infection. (**D**) Percentage of CD4^+^ T cells (top row) or ESAT6-specific CD4^+^ T cells (bottom row) that express PD-1 (left), TIM-3 (center), or both PD-1 and TIM-3 (right) in the lungs of WT or IL-21R^−/−^ mice infected with *M. tuberculosis* for 16 weeks. Data is pooled from 2–4 experiments with a total of 10-23 mice evaluated per group. (**E**) Representative flow cytometric plots showing the expression of PD-1 and TIM-3 by ESAT6-specific CD4^+^ T cells from the lungs of WT or IL-21R^−/−^ 16 weeks after *M. tuberculosis* infection. Each bar or point represents the mean ± SEM *P < 0.05, **P < 0.01, ***P < 0.001, ****P < 0.0001. (**F**) Correlation between the frequency of PD-1^+^TIM-3^+^ TB10.4-specific CD8^+^ T cells and bacterial burden (CFU) in the lungs of WT or IL-21R^−/−^ mice (n = 55 subjects from 5 independent experiments). The relationship between the expression of inhibitory receptors and CFU was different in WT (closed) or IL-21R^−/−^ (open) mice, based on the difference in their slopes determined by linear regression (p < 0.001).

As part of this analysis, we measured the frequency of ESAT6-specific CD4^+^ T cells or TB10.4-specific CD8^+^ T cells in the lungs of WT or IL-21R^–/–^ mice, 16–20 weeks after *M. tuberculosis* infection (Fig. 5a). In 4–5 independent experiments, there were no differences between WT and KO mice with respect to the frequency of these representative antigen-specific CD4^+^ and CD8^+^ T cells. These data emphasize that the evaluation of T cell fitness between genotypes is best done in mixed BM chimeras, which control for differences in local microenvironment and bacterial burden. Furthermore, these data show that in the intact mouse model, there is no difference in the ability of ESAT6-specific CD4^+^ or TB10.4-specific CD8^+^ IL-21R^−/−^ T cells to expand and accumulate in the lungs during infection.

TIM-3 expression by CD8^+^ T cells from WT or IL-21R^−/−^ mice was similar. In contrast, CD8^+^ T cells from IL-21R^−/−^ mice were more likely to express PD-1, and this was true for CD8^+^ T cells expressing both PD-1 and TIM-3 (Fig. 5b). This pattern of PD-1 and TIM-3 expression was even more exaggerated among *M. tuberculosis*-specific CD8^+^ T cells. In WT mice, just 7% of the TB10.4-specific (i.e. tetramer^+^) CD8^+^ T cells expressed PD-1, compared to 59% of the tetramer^+^CD8^+^ T cells from IL-21R^−/−^ mice (Fig. 5b,c). Similarly, the percentage of tetramer^+^CD8^+^ T cells expressing both PD-1 and TIM-3 increased from 5% in WT mice to 45% in IL-21R^−/−^ mice.

While CD8^+^ T cells express more TIM-3 than PD-1 during TB, WT CD4^+^ T cells express more PD-1 (Fig. 5d)^26^. More CD4^+^ T cells from infected IL-21R^−/−^ mice expressed PD-1, TIM-3 or both, compared to WT mice (Fig. 5d,e). Using tetramers, we found that ESAT6-specific CD4^+^ T cells from IL-21R^−/−^ mice were more likely to express PD-1, TIM-3, or both, than CD4^+^ T cells from WT mice. Finally, whether these differences are a consequence of an intrinsic IL-21 signaling, or whether they are secondary to the increased bacterial burden is difficult to discern. However, the magnitude of the effect in IL-21R^−/−^ mice suggests that CFU is not the only factor driving the increased expression of inhibitory receptors (Fig. 5f).

### IL-21 signaling is essential for resistance to TB and T cell mediated protection

**Figure 6:**
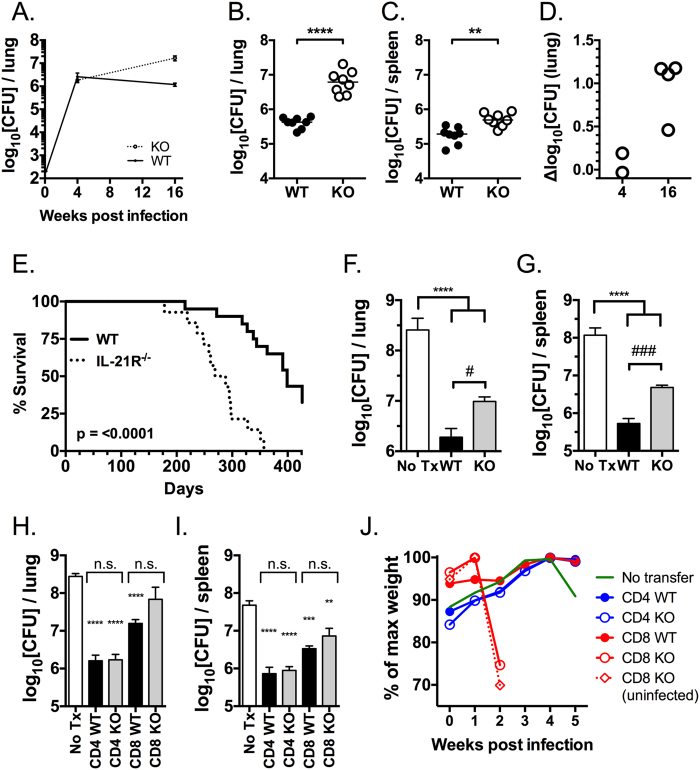
Host resistance to *M. tuberculosis* infection requires IL-21 signaling and is partially mediated through T cell immunity. (**A**) Bacterial burden in the lung from WT (filled circle) or IL-21R^−/−^ (open circle) mice at different time points post infection. The lung (**B**) and spleen (**C**) bacterial burden in WT (filled) or IL-21R^−/−^ (open) mice (n = 8/group), 16 weeks after low dose aerosol infection (d1 inoculum, 44 CFU). **p = 0.01; ****p < 0.0001 by unpaired t test. (**D**) The difference in the lung bacterial burden (Δlog_10_CFU) between WT and IL-21R^−/−^ mice, 4 weeks (2 experiments) or 16 weeks (4 experiments) after low dose aerosol infection (n = 5–8 mice/group). (**E**) Survival of WT (solid line) or IL-21R^−/−^ (dotted line) mice (n = 20 WT; n = 15 KO) after low dose aerosol infection (cumulative data from two independent experiments, each which had a statistically significant difference by the Log-rank (Mantel-Cox) test, p < 0.05). TCRα^−/−^ mice received nothing (open bars, ‘No Tx’), or T cells purified from uninfected WT (black bars) or IL-21R^−/−^ (grey bars) mice. All mice were subsequently challenged with low dose aerosolized *M. tuberculosis*. After four weeks, the bacterial burden in the lung (**F**) and spleen (**G**) were determined. TCRα^−/−^ mice received nothing (open bars, ‘No Tx’), or CD4^+^ or CD8^+^ T cells purified from uninfected WT (black bars) or IL-21R^−/−^ (grey bars) mice. All mice were subsequently challenged with low dose aerosolized *M. tuberculosis*. After four to five weeks, the bacterial burden in the lung (**H**) and spleen (**I**) were determined. In a similar independent experiment, the weights were monitored (**J**). Results are shown for one of two similar experiments. **p < 0.01; ***p < 0.001; ****p < 0.0001, compared to untransferred TCRα^−/−^ mice. n.s., not significant, ^#^p < 0.05; ^###^p < 0.0001 for the comparison of WT vs. IL-21R^−/−^ mice.

Having shown that IL-21 signaling promotes T cell priming, T cell accumulation in the lungs, and cytokine production during TB, we next determined whether IL-21 signaling affects host resistance. A time course showed that after low dose aerosol *M. tuberculosis* infection, WT and IL-21R^−/−^ mice had a similar lung bacillary burden at four weeks (peak immune response) (Fig. 6d); however, by 16 weeks (chronic phase of infection), WT mice had lower lung CFU compared to IL-21R^−/−^ mice (Fig. 6a,b). A similar pattern was observed in the spleen (Fig. 6c). In all, four analyses at week 16 showed that defective IL-21 signaling was associated with a higher pulmonary bacterial burden (Fig. 6d).

The cumulative impact of IL-21 signaling on controlling bacterial growth was assessed by survival. In two independent experiments, WT mice survived significantly longer than IL-21R^−/−^ mice (Fig. 6e). *M. tuberculosis* infection induced qualitatively similar lung lesions in WT and IL-21R^−/−^ mice dominated by macrophages and lymphocytes, and accompanied by small areas of neutrophilic infiltrates and occasional necrotic cells as infection progressed over time. In agreement with the flow cytometric data, the lungs of IL-21R^−/−^ mice contained fewer perivascular and peribronchiolar lymphoid aggregates during early infection when compared to WT mice (Supplemental Fig. 2a). Thus, these data indicate that IL-21 signaling is essential to limit *M. tuberculosis* replication and promote host long-term survival.

An adoptive transfer strategy was used to determine whether IL-21 signaling in T cells promoted host resistance to *M. tuberculosis* infection. WT or IL-21R^−/−^ T cells were obtained from uninfected mice, transferred into TCRα^−/−^ mice, which were then infected with *M. tuberculosis*. Four to five weeks after infection, both groups that received T cells had lower CFU than controls that did not receive T cells, as expected (Fig. 6f,g). Importantly, mice that received WT T cells had lower lung and spleen bacterial loads than those that received IL-21R^−/−^ T cells (Fig. 6f,g). Examination by light microscopy showed that the main beneficial effect of T cell transfer from WT or IL-21R^−/−^ into TCRα^−/−^ recipients was a reduction in necrotizing neutrophilic pneumonia typical of immune deficient hosts, which may reflect macrophage cell death induced by rapid *M. tuberculosis* growth and subsequent neutrophil recruitment (Supplemental Fig. 2b).

We also determined whether there was a difference in the ability of WT or IL-21R^−/−^ CD4^+^ T cells to protect TCRα^−/−^ mice. In contrast to the importance of IL-21 signaling in the transfer of protection by total T cells, WT and IL-21R^−/−^ CD4^+^ T cells transferred equivalent protection (Fig. 6h,i). Protection mediated by transfer of WT or IL-21R^−/−^ CD8^+^ T cells was also determined. Although in one experiment, there was no difference in the ability of the WT and IL-21R^−/−^ CD8^+^ T cells to transfer protection (Fig. 6h, i), this experimental approach was limited by the development of a wasting syndrome in the TCRα^−/−^ mice infused with IL-21R^−/−^ CD8^+^ T cells, which was independent of infection (Fig. 6j).

The finding that IL-21 signaling in total T cells, but not fractionated CD4^+^ T cells is required for transfer of optimal protective immunity against *M. tuberculosis* infection, supports the idea that IL-21 signaling in CD8^+^ T cells contributes to the protection.

## Discussion

IL-21 is a γ_c_ cytokine made predominantly by activated CD4^+^ T cells, which regulates T cell immunity by promoting T cell expansion and CTL function. An established role for IL-21 in chronic LCMV infection^7^,^8^,^9^, together with clinical studies demonstrating IL-21 production during human TB, led us to hypothesize that IL-21 makes an essential contribution to host immunity during *M. tuberculosis* infection.

Limited information exists about IL-21 during experimental *M. tuberculosis* infection. In mice, IL-21 correlates with vaccine efficacy^27^,^28^,^29^, and following primary infection, *M. tuberculosis*-specific CD4^+^ T cells make IL-21 and frequently co-produce IFNγ, IL-2 and/or TNF^30^. A single study measured bacterial growth in IL-21^−/−^ mice following infection, and found comparable bacillary loads in WT and KO mice through day 200 post-infection^31^. Differences in the bacterial strains, initial inoculum, housing conditions, and genetic background of IL-21R^−/−^ vs. IL-21^−/−^ mice could account for the discrepancy with our data. In particular, the virulence of the infecting strain (H37Rv) may be reduced compared to ours (Erdman), since in the same study, a susceptible phenotype was not detected in IL-6^−/−^ mice, which are highly susceptible to *M. tuberculosis*^32^,^33^. These data highlight the importance of strain virulence, inoculating dose, and host genetic background on the outcome of infection in the mouse model^34^.

IL-21R^−/−^ mice have a reproducible susceptible phenotype by week 16. In the absence of IL-21R, *M. tuberculosis* infected mice have a greater pulmonary bacillary load and succumb prematurely. Several cytokines produced by T cells (IFNγ or TNF^35^,^36^), myeloid cells (IL-1, IL-12, TNF, IL-6 32,37,38,39,40) or epithelial cells (GM-CSF^41^) are ascribed crucial roles in host resistance based on the early mortality of knockout mice following infection. In contrast, the mortality of IL-21R^−/−^ mice occurred late during the course of infection (MST = 288 days vs. 399 days, KO vs. WT), which is similar to the accelerated mortality observed in mice lacking CD8^+^ T cells^42^,^43^. Following adoptive transfer of WT or IL-21R^−/−^ TB10Rg CD8^+^ T cells, we tracked T cell priming in the lung-draining LN following infection. We observed that IL-21R^−/−^ cells lag behind WT cells starting 13 days post-infection, indicating that IL-21 signaling promotes efficient CD8^+^ T cell priming. IL-21 signaling appears to have a greater impact in the lung than in the LN. This effect could arise because IL-21 affects ongoing T cell proliferation in the lung with their subsequent accumulation. Alternatively, T cells in the lung may be exposed to greater concentrations of IL-21, either because of the accumulation of activated CD4^+^ T cells producing IL-21, or because of more IL-21 production on a per cell basis. Thus, we argue that IL-21 acts as a ‘Signal 3’ cytokine and is important for initiation of the CD8^+^ T cell responses in the LN. In this way, IL-21 functions similarly to IL-12 by promoting early T cell proliferation and cytokine production^30^,^44^.

Similar to the LN, IL-21R^−/−^ CD4^+^ and CD8^+^ T cells expand poorly in the lung. We used MBMC mice to determine whether IL-21 signaling is required for T cell expansion during infection, a strategy that facilitates the direct comparison of WT and IL-21R^−/−^ T cells in the same inflammatory environment *in vivo*. Four week following aerosol infection, IL-21R^−/−^ T cells are reduced in the blood and lungs relative to WT T cells, and produce less IFNγ and TNF, similar to experiments performed with IL-12R^−/−^ CD8+ T cells^22^. Therefore, IL-21 is a significant driver of T cell immunity to *M. tuberculosis*.

During chronic infection, IL-21R^−/−^ T cells express greater levels of the PD-1 and TIM-3 inhibitory receptors. Higher frequencies of PD-1^+^ T cells are observed in patients with active pulmonary tuberculosis and PD-1 blockade *in vitro* can enhance *M. tuberculosis*-specific IFNγ production^45^. Similarly, TIM-3 blockade *in vivo* during chronic infection improves bacterial control^26^. Since IL-21R^−/−^ T cells already produce less cytokine by week 4, before PD-1 or TIM-3 is significantly expressed, we surmise that IL-21 acts to prevent T cell dysfunction. Although the increased co-expression of PD-1 and TIM-3 by both CD4^+^ and CD8^+^ T cells is a phenotype that is associated with T cell exhaustion during *M. tuberculosis* infection^26^, the increased frequency of PD-1^+^TIM-3^+^ T cells may simply reflect the greater bacillary burden and antigen load in the IL-21R^−/−^ mice. Nevertheless, these data confirm the important role that IL-21 plays in immune-mediated control of *M. tuberculosis* infection.

We have uncovered an essential role for IL-21 in early T cell activation, expansion, and IFNγ production in the mouse model of *M. tuberculosis* infection. IL-21 acts early as shown by the dramatic effect of CD8^+^ T cell priming. In addition, IL-21 appears to have an ‘autocrine’ action and affects CD4^+^ T cell expansion as well. Ultimately, these functions of IL-21 are essential for the long-term maintenance of functional T cell responses during the chronic phase of TB, as loss of bacterial control and a reduction in survival is observed in IL-21R^−/−^ mice. While IL-21 potentially acts on multiple cell types, our data show that a key function of IL-21 is to act on T cells to promote protection. The finding that WT T cells are superior at transferring protection compared to IL-21R^−/−^ T cells supports this conclusion. In contrast to the importance of IL-21R expression by total T cells, there was no difference in the ability of WT or IL-21R^−/−^ CD4^+^ T cells to protect TCRα^−/−^ mice. Although we transferred CD8^+^ T cells into TCRα^−/−^ mice, the experiment is complicated by two factors. First, there is no source of IL-21 since the recipient mice lack both CD4^+^ and NKT cells. Secondly, recipient mice infused with IL-21R^−/−^ CD8^+^ T cells developed a wasting disease necessitating their euthanasia (Fig. 6). The onset of wasting was even more rapid than observed in control TCRα^−/−^ mice and was independent of infection. These factors prevent us from definitively showing that CD8^+^ T cells are a crucial target of IL-21, as has been shown during chronic LCMV infection.

In summary, our data suggest a model in which IL-21 produced by CD4^+^ T cells, promote CD8^+^ T cell expansion and CD8^+^ T cell effector function. In fact, the late mortality of IL-21R^−/−^ mice is typical of mice lacking CD8^+^ T cells or that are defective in effector functions preferentially expressed by CD8^+^ T cells (i.e., perforin). IL-21 has been used therapeutically to treat other chronic infections and cancer and also has the potential to augment vaccine efficacy^46^,^47^. Future experiments should be designed to test these exciting possibilities and determine its role as a correlate of protection.

## Materials and Methods

### Ethics Statement

All methods were performed in accordance with the relevant guidelines and regulations. The animal studies were approved by the Institutional Animal Care and Use Committee at the University of Massachusetts Medical School (Animal Welfare A3306-01), using the recommendations from the Guide for the Care and Use of Laboratory Animals of the National Institutes of Health and the Office of Laboratory Animal Welfare.

### Mice

C57BL/6J, C57BL/6NJ, CD45.1 (B6.SJL-Ptprc^a^Pepc^b^/BoyJ), CD90.1 (B6.PL-Thy1^a^/CyJ), TCRα^−/−^ (B6.129S2-Tcra^tm1Mom^/J), and IL-21R^−/−^ (B6N.129-Il21r^tm1Kopf^/J)^48^, mice were purchased from Jackson Laboratories (Bar Harbor, ME). Mice were 8 to 10 weeks old at the start of all experiments. Mice infected with *M. tuberculosis* were housed in a biosafety level 3 facility under specific pathogen-free conditions at UMMS.

### Generation of mouse bone marrow chimeras

1:1 mixed bone barrow chimeras (MBMCs) were made by lethally irradiating CD90.1^+^ recipients (2 doses of 600 rads separated by three hours). BM was flushed from the femurs, tibia, and humeri of donor mice and RBC lysed. BM cells were then enumerated and groups were combined in a 1:1 ratio. Each recipient mouse received a total of 10^7^ BM cells (5 × 10^6^ of WT and 5 × 10^6^ of KO) via lateral tail vein injection and was kept on antibiotic-treated water for 5 weeks following irradiation. Mice were checked for reconstitution by retro-orbital bleeding to assess the ratio of donor cells in the peripheral blood by flow cytometry. MBMCs were infected with *M. tuberculosis* 8–10 weeks after transfer of the bone marrow cells.

### Generation of retrogenic mice

Detailed information on the generation of TB10_4–11_-specfic retrogenic T cells was previously published^23^. TCR retroviral constructs were generated as 2A-linked single open reading frames using PCR and cloned into a murine stem cell virus-based retroviral vector with a GFP marker as previously described^49^. Retroviral-mediated stem cell gene transfer was performed as previously described^49^. For all experiments shown, the TB10_4–11_-specfic TCR used corresponds to TCR3 (Rg3) as described by Nunes-Alves *et al*.^23^.

### Experimental infection and bacterial quantification

Infection with *M. tuberculosis* (Erdman strain) was performed via the aerosol route, and mice received a day 1 inoculum of 50–200 CFU. A bacterial aliquot was thawed, sonicated once for 30 seconds in a cup horn sonicator, and then diluted in 0.9% NaCl–0.02% Tween 80. The bacterial aliquot was diluted to a final volume of 5 ml, and mice were infected using a Glas-Col aerosol-generation device. To determine CFU, mice were euthanized by carbon dioxide inhalation, organs were aseptically removed, individually homogenized, and viable bacteria were enumerated by plating 10-fold serial dilutions of organ homogenates onto 7H11 agar plates. Plates were incubated at 37 °C and *M. tuberculosis* colonies were counted after 21 days.

### Flow cytometry analysis

Cell suspensions from lung, spleen and lymph nodes were prepared by gentle disruption of the organs through a 70 μm nylon strainer (Fisher) or using the GentleMacs Dissociator (Miltenyi Biotec, Germany) according to the manufacturer’s instructions. For lung preparations, tissue was digested for 30–60 in at 37 °C in cRPMI with 300U/ml collagenase (Sigma) prior to straining. Erythrocytes were lysed using a hemolytic solution (155 mM NH_4_Cl, 10 mM KHCO_3_, 0.1 mM sodium EDTA pH 7.2) and, after washing, cells were resuspended in supplemented RPMI (cRPMI −10% heat inactivated FCS, 10 mM HEPES, 1 mM sodium pyruvate, 2 mM L-glutamine, 50 mg/ml streptomycin and 50 U/ml penicillin, all from Invitrogen) or MACS buffer (Miltenyi Biotec, Germany). Cells were enumerated in 4% trypan blue on a hemocytometer or using a MACSQuant flow cytometer (Miltenyi Biotec, Germany). Surface staining was performed with antibodies specific for mouse CD3 (clone 17A2), CD3ε (clone 145-2C11), CD4 (clone GK1.5), CD8 (clone 53-6.7), CD19 (clone 6D5), CD44 (clone IM7), CD62L (clone MEL-14), CD45.1 (clone A20), CD45.2 (clone 104), CD90.1 (clone OX-7), CD90.2 (clone 53-2.1), CD127 (clone A7R34), KLRG1 (clone 2F1/KLRG1) and Va2 (clone B20.1), (from Biolegend, CA, USA, or from BD Pharmingen, CA, USA). The tetramers of TB10.4_4–11_-loaded H-2 K^b^ and ESAT-6_1–20_-loaded I-A^b^ were obtained from the National Institutes of Health Tetramer Core Facility (Emory University Vaccine Center, Atlanta, GA, USA). All staining was performed for 20 min at 4 °C, unless otherwise stated. Cells were fixed before acquisition with 1% paraformaldehyde in PBS for 60 minutes. Cell analysis was performed on a FACS Canto (Becton Dickinson, NJ, USA) or on a MACSQuant flow cytometer (Miltenyi Biotec, Germany). Data were analyzed using FlowJo Software (Tree Star, OR, USA). For all of the FACS analysis, single-lymphocyte events were gated by forward scatter versus height and side scatter for size and granularity.

### Intracellular cytokine staining

5 × 10^5^−1 × 10^6^ cells were plated in each well of a round bottom 96-well plate and incubated in the presence of TB10.4_4–11_ peptide (10 μM; New England Peptide). Cells incubated in the presence of αCD3/αCD28 (1 μg/mL; BioLegend) or in the absence of stimuli were used as positive and negative controls, respectively. Cells were incubated for 1 hour at 37 °C, at which point GolgiPlug solution (BD Pharmingen, CA, USA) was added to each well for the remaining 4 hours. Cells were collected after a 5 hour stimulation and then surface stained with the antibodies described above, followed by intracellular staining for IFN-γ (clone XMG1.2), TNF (clone MPX6-T22), using the BD Permwash Kit (BD Pharmingen, CA, USA) as per manufacturer’s instructions.

### CD8^+^ T cell priming assay

Single cell suspensions of pools of spleens and lymph nodes from naïve retrogenic mice (6 to 12 weeks post reconstitution) were prepared. CD8^+^ T cells were purified from each suspension using the naïve CD8^+^ T cell isolation kit and magnetic separation (STEMCELL Technologies Inc., Canada). After purification, cells were counted and transferred via the tail vein into congenically marked recipients (CD45.1 or CD90.1), which had been infected 7 days earlier with virulent *M. tuberculosis* (Erdman) via the aerosol route. For all experiments, 2.5 × 10^4^−5 × 10^4^ cells of each group were transferred into each recipient. WT retrogenic CD8^+^ T cells survived in infected mice and were detectable in the lymph nodes, lungs, and spleens for at least 4 weeks after transfer.

### Adoptive T cell transfer

Single cell suspensions of pools of spleens and lymph nodes from naïve WT or IL21R^−/−^ mice were prepared. Total T cells, CD4^+^ or CD8^+^ T cells were purified from each suspension using the respective isolation kit and magnetic separation (STEMCELL Technologies Inc., Canada). After purification, cells were counted and transferred via the tail vein into TCRα^−/−^ mice. The adoptively transferred mice were then infected with virulent *M. tuberculosis* (Erdman) via the aerosol route. For all experiments, 4 × 10^6^−5 × 10^6^ cells of each group were transferred into each recipient.

### Measurement of cell proliferation

For analysis of cell proliferation of retrogenic cells after adoptive transfer, bead-purified naïve Rg cells (see above) were labeled with 5 μM of cell proliferation dye efluor 450 (eBiosciences) in PBS for 20 min at room temperature, followed by extensive washing.

### Cell isolation and microarray analysis

Female C57BL/6 mice were infected with *M. tuberculosis* Erdman as described above. At the indicated time points, mice were euthanized by cervical dislocation and lungs were harvested after perfusion with collagenase containing media. Lungs were allowed to digest in collagenase-containing media for 15 minutes before being homogenized into single cell suspensions. At each time point, the lungs from 3 individual mice were combined into a single sample. T cells were then purified by negative magnetic bead selection (Miltenyi Biotec, Germany). Purified cells were stained to distinguish CD4^+^ and CD8^+^ T cells (CD19, CD3, CD4, CD8). For cell sorting, stained cells were suspended in MACS buffer (Miltenyi Biotec, Germany) and deposited in collection tubes using a BD Aria flow cytometer (Becton Dickinson, NJ, USA). 50,000 CD19^-^CD3^+^CD8^+^ cells were sorted directly into TRIzol Reagent (Life Technologies, California) and immediately frozen. For each time point, samples from 3–4 independent infections were assayed. RNA extraction, microarray hybridization (Affymetrix Mouse Gene 1.0ST array) and data processing were done at the ImmGen Project processing center. Details of the data analysis and quality control can be found at (www.immgen.org).

### Cytokine measurements

Flash frozen lung lobes from infected mice (n = 5 independent subjects) were thawed and lysed using the Bio-Rad Bio-Plex Cell Lysis Kit (Bio-Rad Laboratories, Inc., CA, USA) and a FastPrep-24 homogenizer (MP Biomedicals, CA, USA). Protein concentrations were quantified using the Pierce BCA protein assay kit (Life Technologies, CA, USA) and diluted to a concentration of 1.5 mg/mL in PBS containing BSA. Samples were individually analyzed using the Bio-Rad Bio-Plex Pro Mouse Ctyokine Immunoassay and a Bio-Rad Bio-Plex 200 suspension array reader.

### Histology

Tissue was fixed in zinc formalin, paraffin embedded, sectioned and stained with hematoxylin and eosin as previously described^50^. Microscopic examination was performed by a board certified veterinary pathologist on two serial sections from 5–7 mice per group, focused on the following features: Numbers of inflammatory foci per lung lobe; numbers of lymphoid aggregates per lung lobe in perivascular, peribronchiolar, or intragranuloma locations; relative cellular composition (macrophages, lymphocytes, and neutrophils) of inflammatory foci; and presence or absence of necrosis (inflammatory cells or lung alveolar septae). Digital images were captured magnified 100 or 200 times normal using an Olympus BX41 light microscope fitted with a U-TVO camera and Q-Capture software. The images were minimally processed using Adobe Photoshop CS5.1 to increase brightness and contrast by 10%, and adjust resolution to 300dpi.

### Statistical analysis

All data are represented as mean with SEM. Comparisons of two groups within 1:1 mixed bone marrow chimeras were done with a paired two-sided student’s t-test. All other comparisons were done with an unpaired two-sided student’s t-test and are indicated in the figure legends. Comparisons of more than two groups were done using Holm-Šídák multiple comparisons testing following two-way ANOVA. All tests assume normal distribution of data and the variance was not significantly different between the groups, except for the analysis of the CFU. CFU data were log10 transformed before statistical testing. Significance was represented by the following symbols: The log-rank (Mantel-Cox) test was used to compare survival curves. *P < 0.05, **P < 0.01, ***P < 0.001, ****P < 0.0001, ^‡^P < 0.0001, and N.S. = not significant.

## Additional Information

**How to cite this article**: Booty, M. G. *et al*. IL-21 signaling is essential for optimal host resistance against *Mycobacterium tuberculosis* infection. *Sci. Rep.* **6**, 36720; doi: 10.1038/srep36720 (2016).

**Publisher’s note**: Springer Nature remains neutral with regard to jurisdictional claims in published maps and institutional affiliations.

## Supplementary Material

Supplementary Information
