# Supplementary material for: IL-21 signaling is essential for optimal host resistance against Mycobacterium tuberculosis infection
Source: Sci Rep. 2016 Nov 7;6:36720. doi: 10.1038/srep36720 (PMC5098191; doi:10.1038/srep36720)
Supplement: Supplementary Information [file srep36720-s1.pdf]

IL-21 signaling is essential for optimal host resistance against *Mycobacterium tuberculosis* infection

Matthew G. Booty, Palmira Barreira-Silva, Stephen M. Carpenter, Cláudio Nunes-Alves, Miye K. Jacques, Britni L. Stowell, Pushpa Jayaraman, Gillian Beamer, & Samuel M. Behar

Supplemental Data Set.

Supplemental Figure 1. IL-21 is produced by CD4+ T cells during chronic *M. tuberculosis* infection. $Tim3^{-}PD1^{+}$; $Tim3^{+}PD1^{+}$; $Tim3^{+}PD1^{-}$; and $Tim3^{-}PD1^{-}$ cells sorted from $CD4^{+}$ or $CD8^{+}$ T cells obtained from the lungs of chronically *M. tuberculosis* infected mice were analyzed by Nanostring using a 121 gene codeset as previously reported by our group (Jayaraman et al, 2016, PLoS Pathogens). Shown is the expression data for IL-21, IL-21R, TIM-3 (Havr2), and PD-1 (Pdcd1), for each of those four subsets among (A) $CD4^{+}$ T cells or (B) $CD8^{+}$ T cells. Normalized data from two independent experiments are shown.

Supplemental Figure 2. Histopathological lesions in the lungs of Mtb infected WT and $IL21R^{-/-}$ mice. (A) Accumulation of lymphocytes in M. tuberculosis infected WT and IL21R-/- mice. Lung sections were examined by a board certified veterinary pathologist at 4, 16, and 25 weeks of infection. Lymphoid aggregates in perivascular, peribronchiolar, and intragranuloma locations were identified by the dense clusters of small cells with dark blue to purple nuclei and minimal cytoplasm, and counted manually per lung lobe. Data are shown as the average and standard deviation of the mean of 2 serial lung sections from at least 5 to 7 mice per group per time point. Representative images of WT and IL21R-/- KO lungs with lymphoid aggregates are shown

magnified 100 times normal. Statistical testing was done by a Student’s t-test. (B) Representative images of lung from infected TCRα$^{-/-}$ mice that transferred or not with T cells. Lung lobes from TCRα$^{-/-}$ that received no cells (Left, no transfer), or received an intravenous infusion of naïve WT T cells (WT T cells, middle) or IL21R$^{-/-}$ (KO T cells, right) mice, and were infected with aerosolized Mtb. Four weeks after infection, mice were euthanized and analyzed.

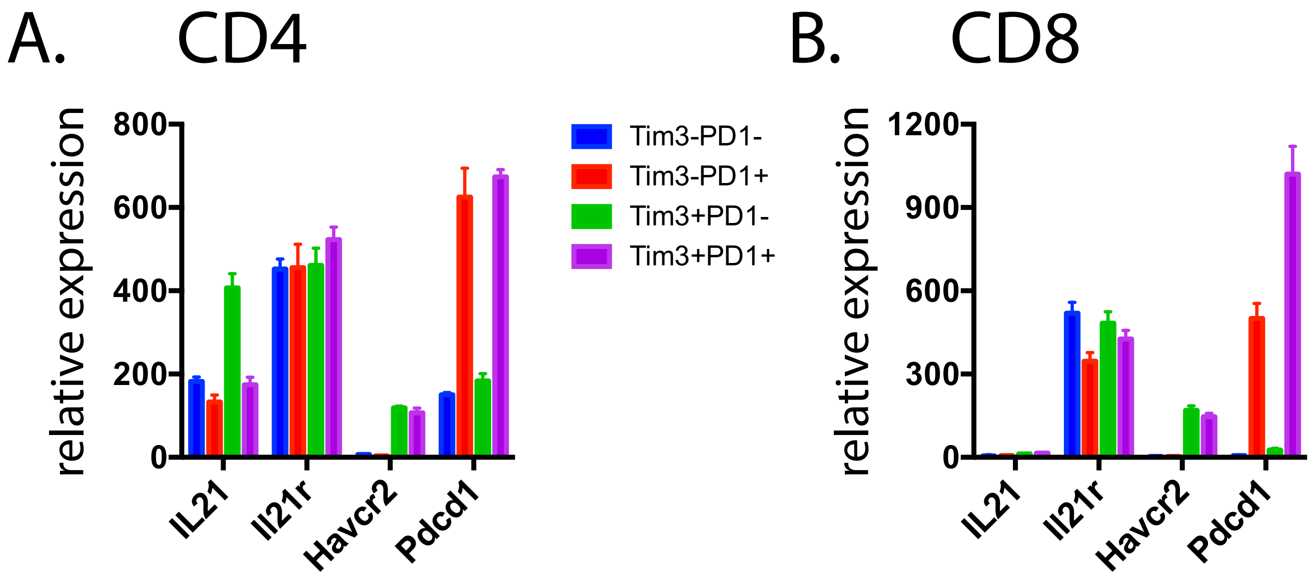

Supplemental Figure 1

A.

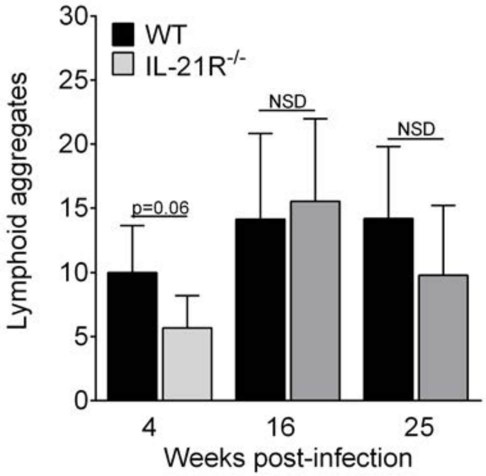

B.

TCRα-/- (No transfer)　WT T cells into TCRα-/-　KO T cells into TCRα-/-

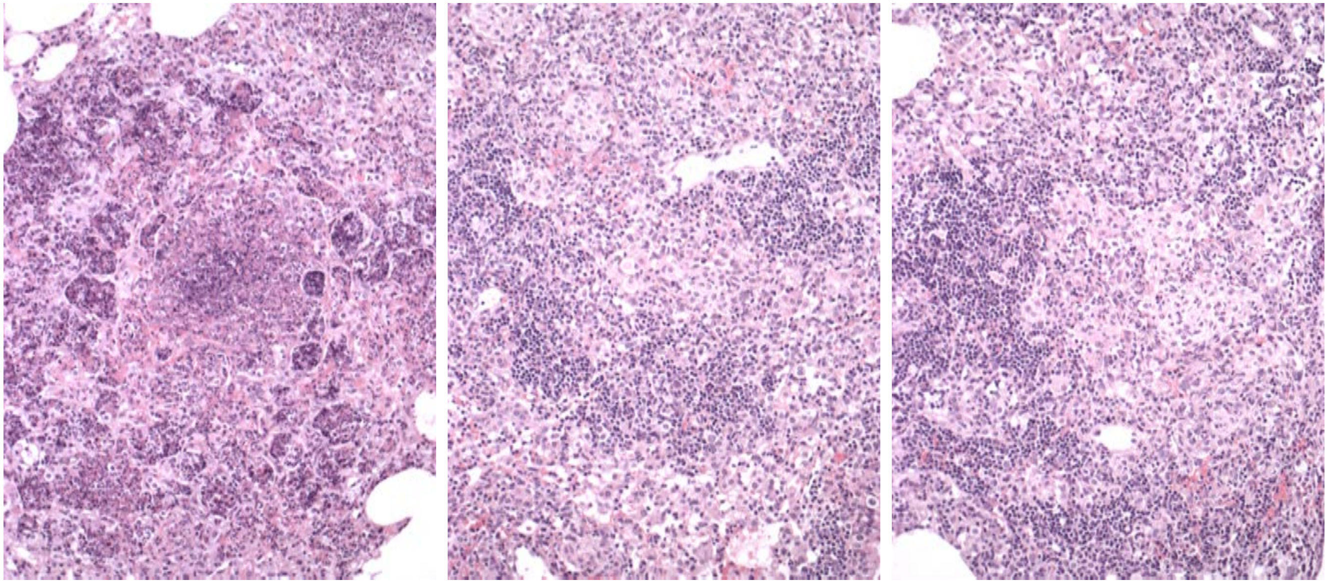

Supplemental Figure 2
